# Is it Possible to Easily Identify Metabolically Healthy Obese Women?

**DOI:** 10.5935/abc.20180228

**Published:** 2018-11

**Authors:** Mauara Scorsatto, Glorimar Rosa, Aline de Castro Pimentel, Ronir Raggio Luiz, Gláucia Maria Moraes de Oliveira

**Affiliations:** 1 Programa de Pós-Graduação em Cardiologia - Universidade Federal do Rio de Janeiro, Rio de Janeiro, RJ - Brazil; 2 Instituto de Nutrição Josué de Castro - Universidade Federal do Rio de Janeiro, Rio de Janeiro, RJ - Brazil; 3 Instituto de Estudos de Saúde Coletiva da Universidade Federal do Rio de Janeiro, Rio de Janeiro, RJ - Brazil

**Keywords:** Cardiovascular Diseases/physiopathology, Metabolic Syndrome, Dyslipidemias, Diabetes Mellitus, Hypertension, Obesity/prevalence, Women

## Abstract

Background: Obesity is recognized as a major risk factor for the development of several metabolic complications. However, some obese individuals have a favorable metabolic profile.

Objective: The aim of this study was to identify an easy parameter for recognizing metabolically healthy obese (MHO) women.

Methods: A total of 292 non-diabetic women with a body mass index (BMI) ≥ 30 kg/m^2^ were selected, and 239 composed the final cohort. We classified the participants according to their metabolic state determined by homeostasis model assessment (HOMA) into MHO or metabolically unhealthy obese (MUO). Both groups were compared regarding biochemical, anthropometric, and body composition characteristics.

Results: The average age of the cohort was 43.9 ± 10.9 years and the average BMI was 37.2 ± 5.3 kg/m^2^. In total, 75.7% of the participants were classified as MHO by HOMA. A cutoff of 108.2 cm for waist circumference (WC) identified MHO participants with a sensitivity of 72.4% (95% confidence interval [CI]: 59.8-82.3%), specificity of 66.9% (95% CI: 59.71-73.3%), and negative likelihood ratio of 0.41 (95% CI: 0.36-0.47). Additionally, a visceral adiposity index cutoff value of 99.2 identified MHO women with a sensitivity of 89.7% (95% CI: 79.2-95.2%), specificity of 48.6% (95% CI: 41.4-55.9%), and negative likelihood ratio of 0.21 (95% CI: 0.15-0.30).

Conclusion: Women classified as MHO exhibited smaller WC measurements and lower body fat percentages, as well as lower blood glucose and insulin levels. WC emerged as an easy parameter for identifying MHO women.

## Introduction

The prevalence of obesity has increased sharply in recent decades. Between 1980 and 2013, it increased by 27% to affect 2.1 billion adults worldwide. A meta-analysis of 97 studies including over 2.88 million individuals and more than 270,000 deaths concluded that obesity is linked to a significantly higher risk of mortality from all causes, including cardiovascular diseases (CVD), when compared with normal weight.^1^ According to recent data, 17% of the Brazilian population over 20 years of age is obese, and women have higher prevalence of diabetes, hypercholesterolemia, and abdominal obesity.^2^

Obesity is recognized as a major risk factor for the development of several metabolic complications. However, some obese individuals have a favorable metabolic profile, characterized by normal homeostasis model assessment (HOMA) index, blood pressure, and lipid profile. These individuals are identified as metabolically healthy obese (MHO),^1^ although there is a current lack of consensus on defining MHO. Recent meta-analysis based in 40 studies showed that almost one-third of obese individuals were MHO using the definition based on the cutoffs established by the Third Report of the National Cholesterol Education Program’s Adult Treatment Panel (NCEP-ATP III) or by those of the International Diabetes Federation (IDF).^3^ Among them, we have Pimentel et al.,^4^ whose studies on Brazilian women showed that around 70% were considered MHO according to HOMA and NCEP-ATP III criteria for the diagnosis of metabolic syndrome.

We hypothesized that individuals with MUO phenotype have increased abdominal adiposity and insulin resistance. Consequently, this study was conducted to identify an easy parameter for detecting MHO women.

## Methods

The sample comprised 239 women recruited in the municipality of São Gonçalo, State of Rio de Janeiro, Brazil. The study was approved by the Research Ethics Committee of the Clementino Fraga Filho University Hospital (Federal University of Rio de Janeiro, Brazil), under certificate number 062/10. All participants signed an informed consent form. The study included women ≥ 20 years of age with a body mass index (BMI) ≥ 30 kg/m^2^. We excluded women who smoked, used drugs or supplements of any kind (including weight loss supplements), were pregnant or nursing, or had pacemakers or metal prostheses (since they would prevent the assessment of body composition by bioimpedance). We also excluded participants who self-reported diagnosis of diabetes mellitus or use of hypoglycemic drugs.

We measured participants’ weight using an electronic scale (Welmy, São Paulo, Brazil). Height was measured using a stadiometer and BMI was calculated as the weight in kg divided by the square of the height in meters. We also measured waist circumference (WC) with a tape measure, body composition with bioelectrical impedance (Biodynamics 450, Seattle, WA, USA), and blood pressure with an aneroid sphygmomanometer (Missouri, Curitiba, Brazil). Finally, in all participants we calculated the waist-to-height ratio (WHtR) in cm/cm. The visceral adiposity index (VAI) was calculated using the following sex-specific formula for women:

$$\mathrm{VAI}\left(\frac{\mathrm{WC}}{36.58+\left(1.89\times \mathrm{BMI}\right)}\right)\times \left(\frac{\mathrm{TG}}{0.81}\right)\times \left(\frac{1.52}{\mathrm{HDL}}\right)$$

Blood samples were collected after a 12-hour overnight fast. Serum was obtained by centrifugation of the samples at 4000 rpm for 15 minutes (Excelsa Baby I, Fanem, São Paulo, Brazil). Serum concentrations of glucose, triglycerides, high-density lipoprotein (HDL)-cholesterol, and total cholesterol were determined by the enzymatic method in an automated biochemical analyzer (LabMax 240, Labtest Diagnostica SA, Brazil). Low-density lipoprotein (LDL)-cholesterol was calculated using the Friedewald formula. Serum insulin was measured by chemiluminescence, and insulin resistance was estimated using the HOMA index.^5^ We distributed the HOMA indices in quartiles and classified the participants as metabolically healthy when their indices were within the three lowest quartiles (2.78), based on Pimentel et al.^4^

The data are presented as mean and standard deviation (SD). The normality of the variables was tested using the Kolmogorov-Smirnov test. Intergroup comparisons were performed with the chi-square test for categorical variables and Student’s t-test for continuous variables. P values < 0.05 were considered statistically significant. We used receiver operating characteristic (ROC) curves to identify the cutoff points for WC and VAI values. The analyses were carried out with the statistical software SPSS 20.0 (SPSS, Chicago, IL, USA).

## Results

We selected 292 women, 53 of whom were excluded after reporting a diagnosis of diabetes mellitus or use of hypoglycemic drugs. The final sample consisted of 239 individuals. A total of 181 participants (75.7%) were classified as MHO according to their HOMA index. The results showed that all anthropometric parameters and VAI were significantly greater in MUO, and that there were fewer hypertensive individuals and higher triglyceride values in the MHO group when compared with the MUO group (Table 1).

**Table 1:** Baseline characteristics of the study participants

	All (n = 239)	MHO (n = 181)	MUO (n = 58)	p value*
Age (years)	43.9 ± 10.9	44.0 ± 10.7	43.6 ± 11.7	0.810
Weight (kg)	93.6 ± 16.0	91.5 ± 15.1	100.2 ± 17.0	< 0.001*
BMI (kg/m^2^)	37.2 ± 5.3	36.3 ± 4.9	39.7 ± 5.5	< 0.001*
Waist circumference (cm)	107.5 ± 11.6	105.4 ± 10.2	114.3 ± 13.3	< 0.001*
Waist/height ratio	67.9 ± 7.1	66.5 ± 6.2	72.1 ± 8.1	< 0.001*
Fat mass (kg)	39.6 ± 9.2	38.2 ± 8.5	44.1 ± 10.1	< 0.001*
Fat mass (%)	41.9 ± 3.3	41.4 ± 3.4	43.2 ± 2.9	< 0.001*
Lean mass (kg)	54.0 ± 7.7	53.2 ± 7.4	56.2 ± 8.2	0.011*
Blood glucose (mg/dL)	99.0 ± 32.7	94.1 ± 24.2	114.4 ± 48.1	0.003*
Insulin (mg/dL)	8.7 ± 7.0	6.0 ± 3.5	17.4 ± 8.0	< 0.001*
Total cholesterol (mg/dL)	200.2 ± 40.9	198.9 ± 3.9	201.9 ± 41.9	0.607
LDL-c (mg/dL)	128.0 ± 39.8	128.9 ± 39.1	124.8 ± 37.3	0.479
HDL-c (mg/dL)	44.5 ± 9.3	44.7 ± 9.6	42.9 ± 9.4	0.193
Triglycerides (mg/dL)	139.0 ± 75.5	128.4 ± 67.2	170.3 ± 89.3	< 0.001*
VAI	133.5 ± 92.0	119.4 ± 81.8	177.4 ± 107.7	< 0.001*
SBP (mmHg)	124.1 ± 19.8	123.5 ± 20.2	126.1 ± 18.7	0.396
DBP (mmHg)	82.7 ± 10.6	81.6 ± 10.8	82.2 ± 10.1	0.671
Skin color - non-whites % (n)	67.4(161)	71.3(129)	55.2(32)	0.064
Marital status - with partner % (n)	60.7(145)	59.7(108)	63.8(37)	0.944
Education ≤ 11 years % (n)	82.9(198)	82.9(150)	82.7(48)	0.918
Income per capita in reais	658.1 ± 524.4	647.6 ± 496.3	691.1 ± 607.6	0.622
Hypertension % (n)	43.9(105)	38.7(70)	60.3(35)	0.004*
Lipid-lowering drugs % (n)	5.0(12)	5.0(9)	5.2(3)	0.952
Hypothyroidism % (n)	5.9(14)	6.6(12)	3.4(2)	0.369
Physical exercise - Yes % (n)	18.8(45)	19.3(35)	17.2(17)	0.722
Menopause - Yes % (n)	34.6(80)	35.6(62)	31.6(18)	0.577

The values are expressed in mean ± standard deviation or frequency (%/n). BMI: body mass index; VAI: visceral adiposity index; SBP: systolic blood pressure; DBP: diastolic blood pressure. To compare the MHO and MUO groups, we used Student's t-test (for continuous variables) or chi-square test (for categorical variables). P value*: statistically significant difference.

**Figure 1:**
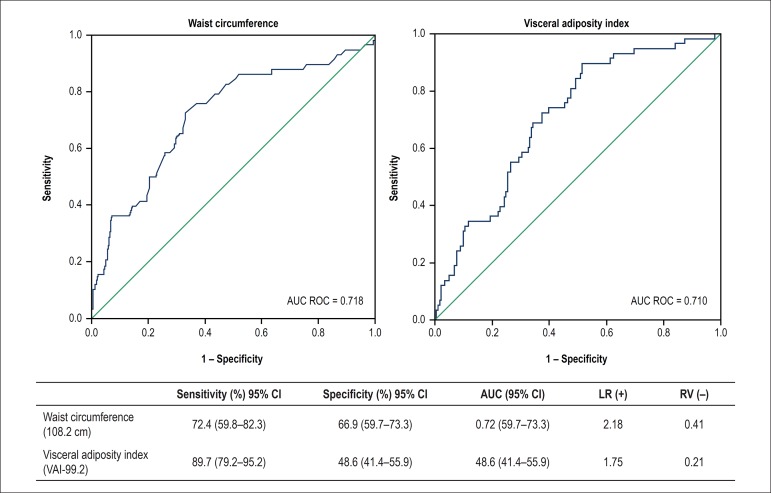
Accuracy and Receiver operating characteristic (ROC) curves for waist circumference and visceral adiposity index at cutoff values of 108.2 cm and 99.2, respectively. LR, Likelihood ratio; AUC: area under the receiver operating characteristic curve; 95% CI: 95% confidence interval; LR: Likelihood ratio; AUC: area under the receiver operating characteristic curve; 95% CI: 95% confidence interval.

Figure 1 shows the values of WC and VAI and their accuracy in identifying MHO women. Both groups presented similar ROC curves; the WC curve had a better negative likelihood ratio to discriminate MHO at a cutoff value of 108.2 cm.

## Discussion

Regardless of the criteria used to define the MHO and MUO phenotypes, it is unclear whether MHO individuals have a lower risk of CVD or all-cause mortality when compared with MUO individuals.^6^ A systematic review of the 14 studies that focused on the risk of CVD showed that most of the studies failed to demonstrate a significant association between MHO and increased risk of CVD and mortality, although MHO individuals may indeed have a slightly increased risk of CVD when compared with individuals with normal weight.^1^^,^^3^

Berezina et al.^7^ studied 503 patients with abdominal obesity and concluded that the MHO phenotype was associated with younger age, smaller WC, higher physical activity level, shorter duration of obesity, and presence of the G45G adiponectin genotype.^7^ However, the greatest challenge is establishing a cutoff point for WC that can be applied to different obese populations.

In our study, the prevalence of metabolic health was high; approximately 76% of obese individuals were MHO, and these results were influenced by which definition of metabolic health was used. According to those results, increased waist circumference, waist-to-height ratio, fat mass, blood glucose, insulin, triglycerides, VAI, and hypertension were associated with the MUO phenotype, suggesting that the criterion applied could identify individuals with higher CVD risk. This phenotype overlaps the so-called hypertriglyceridemic waist phenotype, associated with atherosclerosis, diabetes, and coronary artery disease.^1^^,^^3^^,^^6^ Also, this higher prevalence of MHO suggests lack of evidence that BMI is a good marker of cardiometabolic risk and that there is a need for the development and validation of other markers that may help to guide diagnosis and treatment of obese individuals.^7^

In a recent study,^8^ including 296,535 participants of both sexes from the UK Biobank followed up for an average of 5 years, one standard deviation increase in waist circumference (12.6 cm for women and 11.4 cm for men) was associated with a hazard ratio (HR) of 1.16 (95% CI: 1.13-1.19) for women and 1.10 (95% CI 1.08-1.13) for men for CVD events. In our study, WC had greater measurement values and was an inexpensive and easy tool to apply in a clinical setting in order to discriminate Brazilian women with MHO from those with MUO. Also, WC and VAI identified MHO women with a similar area under the ROC curve.

The VAI was a positive independent indicator of arterial stiffness, measured by brachial-ankle pulse wave velocity in 5,158 individuals over the age of 40 in a cross-sectional study conducted in Nanjing, China.^9^ However, VAI is not so easily obtained in clinical practice. It is possible that WC and VAI could be markers of different aspects of MHO. The former is a tool that easily identifies MHO individuals, and the later assesses the effects of obesity on arterial stiffness and transition into an unhealthy state.

Hamer et al.^10^ followed up 2,422 men and women for over 8 years as part of the English Longitudinal Study of Ageing. These authors showed that the MHO phenotype is relatively unstable, since 44.5% of MHO individuals transitioned into an unhealthy state, and emphasized that the progress to an unhealthy state was linked with a significant increase in WC.^10^ Visceral obesity is associated with pro-inflammatory activity and increased production of adiponectin linked to deterioration of insulin sensitivity, increased risk of diabetes, dyslipidemia, hypertension, atherosclerosis, and higher mortality.^10^

The primary issue is that the number of obese individuals is continually increasing, and it would be unaffordable to treat all of them in the same fashion. When it comes to obese individuals, as a rule, they all exhibit higher WC measures than the values proposed as cutoff points by IDF and NCEP-ATP III.^3^ In our study, there is a lack of information regarding some other variables that have been used to define MHO, such as production of adiponectin and inflammatory markers. The strengths of this study include the sample size and the study setting. Furthermore, by easily identifying high-risk obese individuals, this study may make lifestyle modification possible.

There has been much interest in the paradoxical findings of individuals considered MHO despite increased adiposity. The major challenge was to determine a single parameter for detecting MHO women, given that there is no consensus in literature and that few studies have been conducted in Brazil. Therefore, our study suggests that waist circumference is an easy parameter for identifying MHO women.
